# Electrocatalytic CO_2_ Reduction in Acids: A Groundbreaking Approach to Converting CO_2_ into Fuels and Feedstocks

**DOI:** 10.34133/research.0589

**Published:** 2024-01-13

**Authors:** Wenbo Wei, Haifei Liu, Qi-Long Zhu

**Affiliations:** ^1^ School of Materials Science and Engineering, Zhejiang Sci-Tech University, Hangzhou 310018, China.; ^2^ State Key Laboratory of Structural Chemistry, Fujian Institute of Research on the Structure of Matter, Chinese Academy of Sciences (CAS), Fuzhou 350002, China.; ^3^ University of Chinese Academy of Sciences, Beijing, 100049, China.

## Abstract

The electrocatalytic carbon dioxide reduction reaction (CO_2_RR) at industrial-level current densities provides a sustainable approach to converting CO_2_ into value-added fuels and feedstocks using renewable electricity. However, the CO_2_RR conducted typically in alkaline and neutral electrolytes encounters some challenges due to the inevitable reaction between CO_2_ and OH^−^ ions, which undermines CO_2_ utilization and leads to poor operational stability. Acidic media present a viable alternative by reducing (bi)carbonate production, thereby enhancing the carbon efficiency and stability in CO_2_RR. The objective of this paper is to provide a concise account of the recent advancements and challenges in the field of acidic CO_2_RR, with an emphasis on future developments and opportunities.

Converting carbon dioxide (CO_2_) into hydrocarbon fuels and chemicals offers a promising approach to CO_2_ utilization, advancing carbon-negative solutions [1–3]. Particularly, the electrochemical CO_2_ reduction reaction (CO_2_RR) exhibits considerable promise for industrial implementation, due to advantages, including the use of renewable electricity, mild and safe operating conditions, and relatively straightforward and clean processes, allowing for the generation of a diverse array of reduction products (including C_1_ [CO, HCOOH, CH_4_, etc.] and C_2+_ [C_2_H_4_, C_2_H_5_OH, C_3_H_8_, etc.]) [4–8]. However, the practical applications of CO_2_RR in alkaline and neutral systems are still hindered by severe disadvantages. First, in alkaline media, a considerable proportion (>50%) of the input CO_2_ reacts with OH^−^ to form (bi)carbonate, which ultimately results in low carbon efficiency and negative energy balance. Second, the accumulation and precipitation of (bi)carbonate and electrolyte flooding in the cathodic gas-diffusion electrode inevitably result in poor operational stability (Figure). Third, the anion-exchange membranes for CO_2_RR still suffer from a stability issue and low ion conductivity at high pH.

**Figure.**
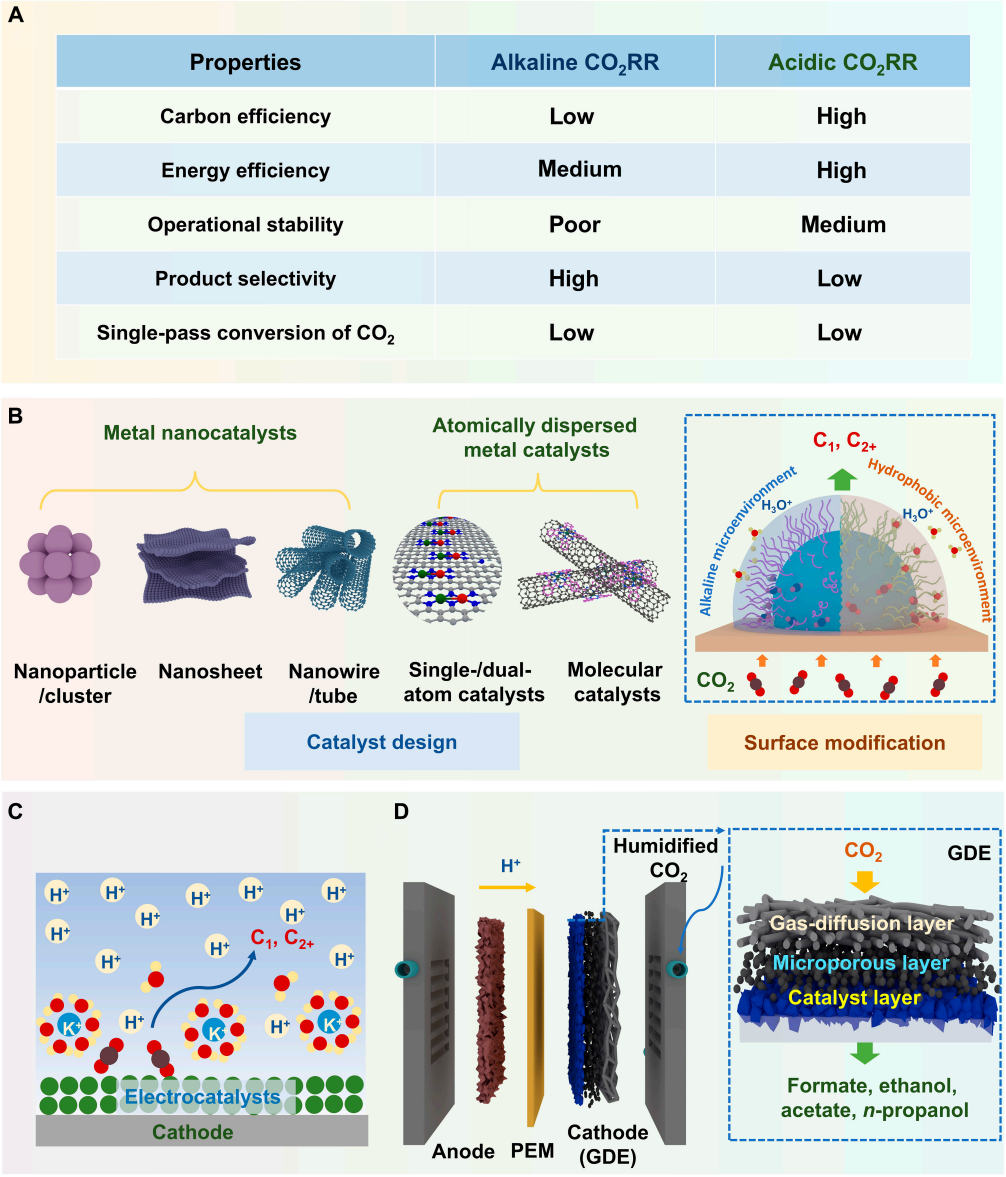
Acidic CO_2_ reduction reaction (CO_2_RR): (A) comparison between alkaline and acidic CO_2_RR; (B) electrocatalysts; (C) electrode–electrolyte interface design; (D) schematic of a gas-diffusion electrode (GDE)-integrated membrane electrode assembly (MEA) cell. PEM, proton-exchange membrane.

Given these challenges, many researchers are focusing on acidic CO_2_RR owing to its significant advantages (Figure), particularly higher carbon efficiency, more stable operation, and lower energy requirements, as compared to the alkaline one [9,10], which are garnering growing interest for potential industrial applications. Furthermore, the proton-exchange membranes used in acidic CO_2_RR can offer excellent proton conductivity and stability. However, the competitive hydrogen evolution reaction (HER) is augmented under acidic conditions, resulting in a diminished selectivity for CO_2_RR [11]. Meanwhile, the formation of C_2+_ products might be harder, limiting the product range in acidic conditions. In addition, some catalysts can suffer from corrosion at high potentials in acidic media. To tackle these issues, extensive research has been conducted on the design and synthesis of efficient catalysts, the development of practical electrolytic devices, and the investigation of reaction mechanisms.

A thorough understanding of CO_2_RR and HER mechanisms in acidic media is essential for optimizing CO_2_RR while mitigating HER. In acidic media, CO_2_RR is facilitated by the rapid diffusion of CO_2_, restrained migration of H^+^ and H_2_O to active sites, and local confinement of OH^−^ ions. However, the rise of proton concentration in acidic media accelerates HER, greatly decreasing the Faradaic efficiency (FE) of CO_2_RR. In particular, an acidic environment can impede the availability of local intermediates and the subsequent C–C coupling, thereby restricting the conversion of CO_2_ to C_2+_ products [12]. Consequently, it is essential to rationally design an advanced electrocatalytic system with highly active and stable catalysts, which could optimize the adsorption energy barriers of CO_2_ and intermediates at active sites, promote CO_2_ diffusion while limiting the transport of H^+^ and H_2_O to the active sites, and synergistically establish a localized microenvironment to promote the acidic CO_2_RR.

To improve the activity and selectivity of CO_2_RR in acidic media, the exploration of advanced electrocatalysts is an essential prerequisite. On the one hand, modifying the composition, coordination environments, and nanostructures of the catalysts can effectively modulate the electronic structures of active sites. Optimal electrocatalysts should exhibit appropriate adsorption for reactants and intermediates (e.g., *CO_2_, *CO, *COOH, *CHO, and *OCHO) [11,13,14], thus improving the acidic CO_2_RR while suppressing HER. For instance, Li et al. [13] identified that electron transfer from Cu donors to Bi acceptors in bimetallic Cu–Bi nanosheets could potentially enhance the acidic CO_2_RR. On the other hand, the use of functional ligands to modify the catalyst surface enables the creation of local microenvironments that could regulate interfacial wettability, provide noncovalent interactions, stabilize intermediates, and more (Figure). For instance, Zhang et al. [15] devised a general strategy that can alter the mass distribution surrounding the active sites, by incorporating quaternary ammonium functional groups with extended alkyl chains into the molecular catalysts. In this system, the stable cationic layer stabilizes negatively charged *CO_2_^−^ intermediates while repelling hydrogen ions, and the long alkyl chains adjust the interfacial environment for deterring water molecules, thus inhibiting HER. In addition, given the inherent instability of most catalysts in acidic environments, it is highly desired to develop catalysts that demonstrate high stability in acidic media. For instance, Fang et al. [14] reported an excellent pH-tolerant, low-cost, and recycled lead electrocatalyst obtained from lead-acid battery waste, for reducing CO_2_ to formic acid with a high FE over 91%, which can operate continuously for more than 5,200 h at a cell voltage of 2.2 V with a current density of ~600 mA cm^−2^.

The choice of electrolytes that directly interact with the active sites, reactants, intermediates, and products also exerts a significant influence on the efficiency, selectivity, and durability of the acidic CO_2_RR. It has been found that the introduction of cation species into acidic electrolytes has been identified as an effective approach for limiting proton mass transport to the electrode surface, which in turn enhances the CO_2_RR activity and selectivity while inhibiting the HER (Figure). Zhang et al. [16] revealed the mechanism of alkali-cation-enhanced CO_2_RR on Cu in acidic media by in situ spectroscopy characterizations. It was verified that the flexible water networks around larger cations (e.g., K^+^) facilitate water reorientation and the proximity of hydrogen to CO_2_, thus boosting CO_2_RR.

Furthermore, the construction of advanced electrode configurations can effectively enhance interfacial mass transfer, reduce electrolyte resistance, and augment the stability of the system, which is also crucial for the improvement of acidic CO_2_RR performance [17]. In particular, gas-diffusion-electrode-integrated membrane electrode assembly cells, known as “zero-gap” and “catholyte-free” for gas reactant electrolysis, can deliver gaseous CO_2_ directly to the surface of the electrocatalysts (Figure), thereby overcoming the limitations of solubility and mass transfer in aqueous electrolytes relative to classical flat electrodes and H-type cells [18–20]. In a typical example, Pan et al. [20] designed an acid-fed membrane electrode assembly for CO_2_ reduction to CO, achieving a high FE over 80% in an electrolyte solution comprising 0.01 M H_2_SO_4_ and 0.01 M Cs_2_SO_4_, with a single-pass conversion efficiency of approximately 90%.

The acidic CO_2_RR presents a promising avenue for the direct conversion of CO_2_ into high-value chemicals and fuels and offers an efficacious approach to advancing the industrial implementation of CO_2_RR. Despite significant advancements in the exploration of acidic CO_2_RR, including crucial developments in the catalyst preparation and the regulation of catalytic microenvironments, electrode structures, and electrolytes, this field still encounters numerous challenges: (a) The high acidity can promote HER and accelerate catalyst corrosion. Although the addition of alkali ions hinders proton migration, it easily leads to salt precipitation during long-term electrolysis. Thus, in addition to the intrinsic activity enhancement, the surface modification of the catalysts could be paid more attention, which may not only construct a favorable microenvironment for CO_2_RR and promote the performance toward multicarbons but also enhance the stability of the catalysts in acidic media. (b) The catalysts may experience complex dynamic reconstruction due to corrosion and redeposition processes in acidic environments. Therefore, a comprehensive understanding of catalytic interfaces must be further enhanced through in situ techniques, such as transmission electron microscopy, x-ray absorption spectroscopy, Fourier transform infrared spectroscopy, Raman spectroscopy, and x-ray diffraction. (c) In acidic media, CO_2_RR exhibits reduced selectivity for the target products, particularly for C_2+_ products. Therefore, a deeper understanding of the reaction mechanism through multiple characterizations is necessary for developing more effective catalytic CO_2_RR processes in acidic media. (d) In addition, in acidic CO_2_RR, the anodic oxygen evolution reaction has slow kinetics, leading to a large overpotential and high overall energy input. Notably, the substitution of oxygen evolution reaction with a more thermodynamically favorable organic oxidation reaction, which can even generate value-added chemicals at much lower potentials, represents a promising avenue for further investigation. (e) It is evident that laboratory-scale electrolyzers for acidic CO_2_RR are inadequate for industrial applications. The development of electrolysis equipment with a low ohmic loss, long-term stability, and scalability is essential for facilitating the transition to large-scale applications. The single-pass conversion of CO_2_, a key performance metric for practical implementation, is typically below 20% at high current densities, which should be notably improved through reactor design and flow optimization. Furthermore, establishing a standardized evaluation system to assess the performance and economic viability of acidic CO_2_RR is imperative for promoting the industrial adoption of this technology.
